# Reporting of Perirenal Hematoma Size After Ultrasound-Guided Renal Biopsy in Adults: A Scoping Review

**DOI:** 10.3390/biomedicines13122943

**Published:** 2025-11-29

**Authors:** Piotr Białek, Weronika Banasik, Adam Dobek, Michał Żuberek, Krzysztof Falenta, Ilona Kurnatowska, Ludomir Stefańczyk

**Affiliations:** 1 1st Department of Radiology and Diagnostic Imaging, Medical University of Lodz, Kopcinskiego 22 Street, 90-153 Lodz, Poland; 2 Department of Internal Diseases and Transplant Nephrology, Medical University of Lodz, Kopcinskiego 22 Street, 90-153 Lodz, Poland

**Keywords:** perirenal hematoma, renal biopsy, ultrasound, bleeding complications, chronic kidney disease, scoping review

## Abstract

**Introduction:** Percutaneous renal biopsy (PRB) is the gold standard for diagnosing nephropathies, but it carries a risk of bleeding complications, mainly perinephric hematomas (PHs). While PH incidence is often reported, the significance of PH size remains insufficiently explored. This scoping review systematically mapped the evidence on PH size after ultrasound-guided PRB in adults, focusing on imaging modalities, measurement methods, the definition of ‘large’ PH, factors influencing PH size, and its clinical implications. **Materials and Methods:** Following the Joanna Briggs Institute methodology, we searched PubMed/MEDLINE, Embase, Cochrane CENTRAL, and Scopus through 27 August 2025. Eligible studies included at least 50 adult subjects undergoing ultrasound-guided PRB with quantitative, imaging-based assessment of PH size. **Results:** Fifty-one studies met the inclusion criteria. Almost all relied on ultrasound, with only one using computed tomography. PH size was measured using heterogeneous methods, most often one-dimensional diameters, less frequently surface area or volumetry, with no standardization. Reported PH frequencies varied substantially across studies (1.1–85%), likely reflecting differences in imaging protocols, timing, and reporting thresholds. Several studies proposed PH size thresholds (e.g., diameter ≥ 2–3 cm, volume ≥ 40–85 mL) linked to adverse outcomes such as transfusion or hemodynamic instability. Factors associated with larger PHs included needle gauge, number of passes, impaired kidney function, coagulopathy, and certain histopathologies. **Conclusions:** PH size has prognostic value beyond incidence alone. Standardized measurement and reporting are needed to clarify its clinical relevance after PRB.

## 1. Introduction

### 1.1. Background and Rationale

Percutaneous renal biopsy (PRB) with subsequent histopathological evaluation is the gold standard for the diagnosis of nephropathies [1,2,3,4]. Since its introduction in the mid-20th century [1,5,6,7,8,9], the procedure has undergone substantial evolution—from blind techniques to approaches guided by imaging [1,9,10,11], with ultrasound (US) being the standard of care [3,7,12]. The ability to directly assess glomerular, interstitial, and vascular structures has made PRB an indispensable tool in clinical practice, guiding diagnosis, prognosis, and therapeutic decisions [1,7,11].

Despite being considered a safe procedure, PRB is not entirely free from complications [3,7,9,11,12,13]. The most common are bleeding events, particularly hematuria and perirenal hematomas (PH) [3,6,7,8,11,12,13,14,15]. Reported incidence rates of PH vary widely across studies [3,6,9,12,14,15,16]. This heterogeneity may result not only from patient- and technique-related differences [8], but also from methodological aspects—whether PH were systematically sought in all patients or only in symptomatic individuals, and at what interval after PRB imaging was performed [6,7,9,15]. Furthermore, some studies suggest that small PHs may occur in the vast majority of patients after PRB, often without clinical consequences [3,6,7,9,15]. This observation limits the interpretative value of frequency alone and raises the question of whether PH size and other characteristics may also be relevant for clinical outcomes [3].

**Figure 1:**
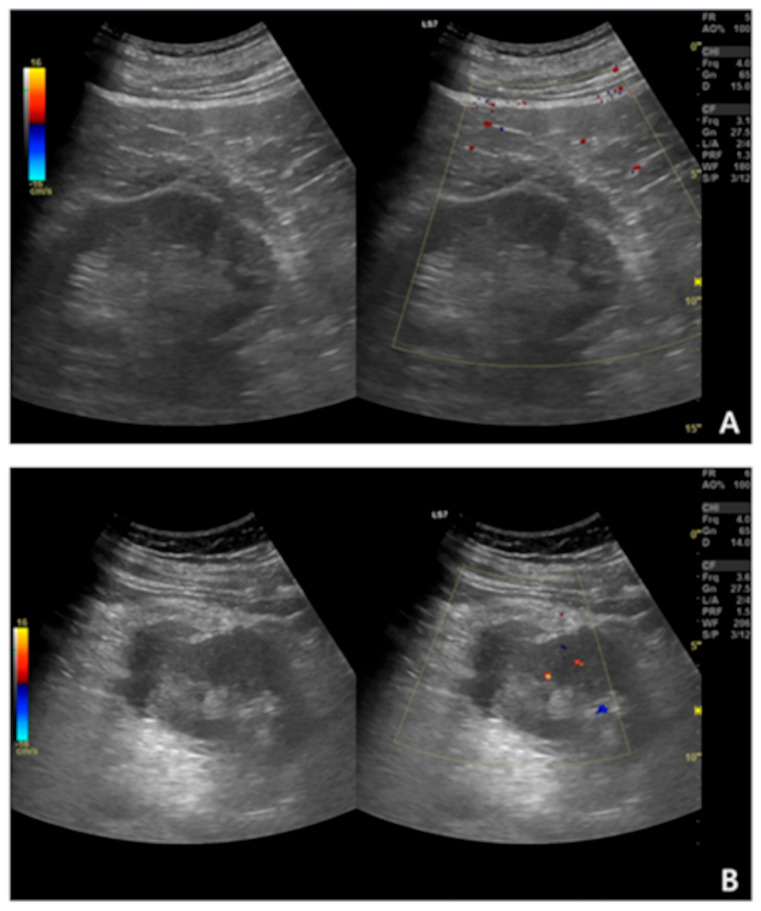
Ultrasound (with Color Doppler) images of post-biopsy perinephric hematomas. Images (**A**,**B**) show hematomas that differ in echogenicity and morphology, illustrating the variability of their sonographic appearance and the challenges of reliably assessing perinephric hematomas on ultrasound.

Most authors primarily rely on clinical observation and laboratory monitoring (e.g., hemoglobin (Hb) or hematocrit levels, urinalysis) to detect complications, which appears to be a pragmatic approach [7,9,16]. Basic biochemical parameters such as Hb, platelet count, and coagulation indices (activated partial thromboplastin time (APTT), prothrombin time (PT), international normalized ratio (INR)) are routinely checked to assess bleeding risk, and a post-biopsy drop in Hb is often used as a marker of clinically relevant hemorrhage. However, these laboratory findings are rarely analyzed in direct relation to PH size. Nonetheless, imaging may provide additional clinically relevant insights [17,18]. US is the most widely used modality due to its accessibility and safety, and its role in post-PRB monitoring is well established [6,7,16]. Figure 1 illustrates the sonographic appearances of post-biopsy PHs.

Against this background, a scoping review is warranted to systematically map the existing evidence on PH size in adult patients. The review aimed to establish how studies report PH size, how PHs are measured with different modalities, how ‘large’ and ‘small’ PHs are defined, and what clinical implications are attributed.

Unlike prior systematic reviews that focused primarily on the incidence of bleeding complications after renal biopsy, the present scoping review specifically maps how PH size is measured, quantified, and reported across studies. The focus on measurement methodology, rather than incidence, represents the principal added value of our work and, to our knowledge, has not been previously synthesized.

### 1.2. Research Questions

The primary question of this scoping review was how the size of PH after PRB (US-guided) is reported in imaging-based studies. The secondary question focused on what definitions of ‘small’ and ‘large’ PH were applied and what results and conclusions regarding their occurrence and clinical consequences were reported.

## 2. Materials and Methods

### 2.1. Scoping Review

This scoping review was conducted in accordance with the Joanna Briggs Institute (JBI) methodological guidelines [19,20] and reported following the PRISMA-ScR (Preferred Reporting Items for Systematic reviews and Meta-Analyses extension for Scoping Reviews) statement [21]. The review protocol was not registered in PROSPERO or any other database.

### 2.2. Ethics

As this review was based exclusively on previously published studies and did not involve any primary patient data, approval from an ethics committee was not required.

### 2.3. Search Strategy

A comprehensive literature search was carried out on 27 August 2025. An initial limited search was performed in four databases (PubMed/MEDLINE, Embase, Cochrane Central Register of Controlled Trials [CENTRAL], and Scopus) to identify relevant keywords and controlled vocabulary terms (MeSH in PubMed, Emtree in Embase). Titles, abstracts, and index terms of retrieved articles were examined to refine search terms. The finalized strategy was subsequently applied systematically across all four databases to ensure broad coverage of the nephrology and radiology literature.

The search strategy combined three conceptual blocks: (1) renal biopsy (e.g., ‘renal biopsy’, ‘kidney biopsy’, ‘percutaneous renal biopsy’, ‘percutaneous kidney biopsy’), restricted to percutaneous procedures performed under ultrasound guidance; (2) hematomas and bleeding complications (e.g., ‘hematoma’, ‘hemorrhage’, ‘bleeding complication’); and (3) imaging modalities used for the detection of post-biopsy hematomas (e.g., ‘ultrasonography’, ‘computed tomography’, ‘magnetic resonance imaging’). Both controlled vocabulary (MeSH in PubMed, Emtree in Embase) and free-text terms (searched in titles and abstracts with synonyms included to maximize sensitivity) were employed. Boolean operators (AND, OR, NOT) were used to combine the search blocks. No date restrictions were applied, and only English-language studies were considered. Full, database-specific search strategies for all four databases (PubMed/MEDLINE, Embase, CENTRAL, Scopus) are provided in Supplementary Table S1 to ensure full transparency and reproducibility.

Reference lists of included studies were screened to identify additional articles. Grey literature was not searched.

### 2.4. Eligibility Criteria

#### 2.4.1. Population

- Adult patients (≥18 years) who underwent PRB, including both native and transplant kidneys.
- Excluded: pediatric populations (<18 years), unless data for adults were clearly extractable; biopsies performed for the diagnosis or suspicion of renal tumors; studies conducted in animals or cadavers.
- Studies with mixed populations (e.g., adults and children, or different biopsy types) were included only if relevant data for adult patients undergoing US-guided PRB could be separated; otherwise, such studies were excluded.

#### 2.4.2. Concept

- Studies that reported imaging-based assessment of PH size after biopsy. Eligible studies quantified PH size using explicit numerical measures (e.g., thickness, cross-sectional area, volume or size distribution expressed in quantiles such as tertiles or quartiles). Studies that classified PH size only in qualitative or arbitrary terms (e.g., ‘small’ vs. ‘large’) without providing numeric data were excluded.
- All imaging modalities were accepted.
- Excluded: studies without imaging-based PH assessment, studies relying solely on clinical criteria without imaging confirmation, or not addressing biopsy-related complications.
- Additional information on risk factors or clinical consequences was extracted if available.

#### 2.4.3. Context

- PRB performed under US guidance in any clinical setting.
- Excluded: blind biopsies (without imaging guidance), open surgical kidney biopsies, transjugular biopsies, and procedures guided exclusively by CT or other imaging modalities.
- No geographic restrictions were applied.

#### 2.4.4. Types of Studies

- Eligible: original research articles with primary data, including observational (prospective or retrospective), interventional studies, and cohort analyses with at least 50 participants
- Excluded: case reports, case series, reviews, meta-analyses, commentaries, letters, conference abstracts without full text, grey literature, and other secondary or incomplete sources.
- Studies available only as abstracts without full text, or lacking an abstract, were excluded.

#### 2.4.5. Language and Timeframe

- Only studies published in English were considered.
- No date restrictions were applied; all available literature was included.

### 2.5. Definitions

There was substantial heterogeneity in terminology across the studies we reviewed. Some authors referred to ‘perinephric hematoma’ [22,23]; others used terms such as ‘perirenal hematoma’ [4,24], ‘renal hematoma’ [25], ‘pararenal hematoma’ [26], ‘subcapsular hematoma’ [2], ‘subcapsular perinephric’ [11,27] ‘retroperitoneal hemorrhage’ [28] or some combined terms such as ‘subcapsular perinephric’ [11,27,29], ‘parenchymal + perirenal’ [15], or simply reported ‘hematoma’ [30,31], ‘bleed’ [31], or ‘bleeding’ [32]. As a result, many studies did not provide an explicit definition, while others either distinguished between these entities or grouped them together.

To ensure consistency in our review, all these terms were treated uniformly and categorized under the overarching concept of ‘perinephric hematoma’ (PH). This approach reflects the fact that in the studies included in this research, most did not apply strict definitions, and even when differences were acknowledged, the terms were often used interchangeably.

### 2.6. Study Selection and Data Charting

**Figure 2:**
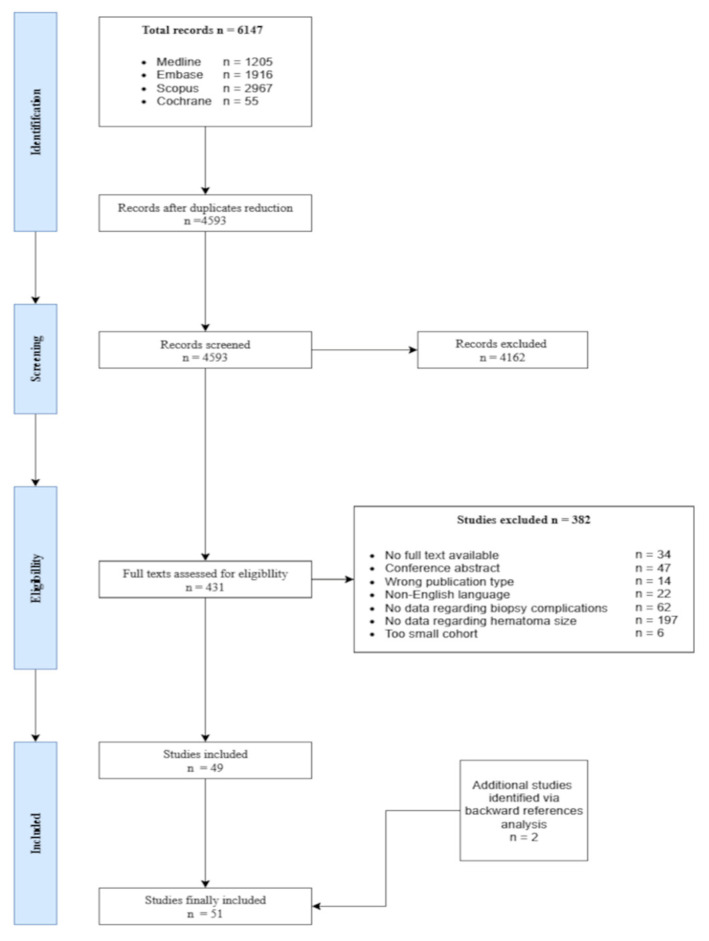
Flowchart illustrating the selection process of studies on perinephric hematomas after ultrasound-guided percutaneous renal biopsy. The diagram shows the phases of identification, screening, eligibility, and inclusion, resulting in 51 studies included in the review. The figure was prepared in accordance with the Preferred Reporting Items for Systematic Reviews and Meta-Analyses extension for Scoping Reviews (PRISMA-ScR) as described by Tricco AC et al., Annals of Internal Medicine, 2018, pp. 467–473. doi: https://doi.org/10.7326/M18-0850 [21].

All search results were imported into Rayyan (Rayyan Systems Inc., Doha, Qatar) for reference management and deduplication. Study selection was performed in two stages: first, titles and abstracts were screened independently by two reviewers, followed by full-text assessment of potentially eligible studies. Any discrepancies were resolved through discussion until consensus was reached. The selection process is summarized in the PRISMA-ScR flow diagram (Figure 2).

Data were charted using a standardized Excel form, piloted and refined during the process. Two reviewers independently extracted bibliographic details, study design, setting, population characteristics, imaging method and timing, PH occurrence, definitions of ‘small’ and ‘large’ PH, and reported results and conclusions. Discrepancies were resolved by consensus.

Data were synthesized narratively, focusing on the descriptive summary of study characteristics and hematoma size assessment.

## 3. Results

The database search retrieved 6147 records (Medline n = 1205, Embase n = 1916, Scopus n = 2967, Cochrane n = 55). After duplicates were removed, 4593 records underwent screening of titles and abstracts, leaving 431 full texts for detailed evaluation. Among these, 382 articles were excluded due to lack of full text (n = 34), conference abstract only (n = 47), inappropriate publication type (n = 14), non-English language (n = 22), no data on PRB complications (n = 62), no information on PH size (n = 197), or a cohort considered too small (n = 6). In total, 49 studies fulfilled the criteria, and two additional studies were identified through reference checking, resulting in 51 studies being included in the review (Figure 2).

**Figure 3:**
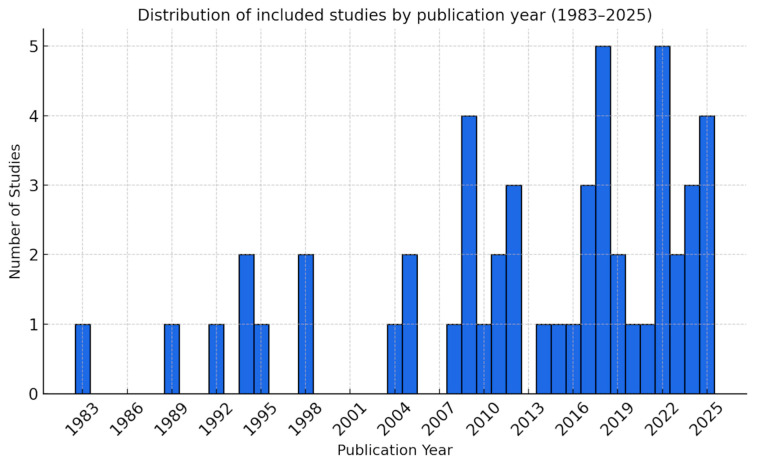
Histogram of included studies by year of publication (1983–2025). The figure demonstrates a gradual increase in research output over time, with peaks in 2009, 2018, 2022, and 2025. Most studies were published within the last decade.

Studies included [3,4,11,15,18,22,23,24,25,26,27,28,29,30,31,32,33,34,35,36,37,38,39,40,41,42,43,44,45,46,47,48,49,50,51,52,53,54,55,56,57,58,59,60,61,62,63,64,65,66,67] in the scoping review are presented in Table 1, while more detailed methodological and imaging-related characteristics are provided in Supplementary Table S2. The included studies were published between 1983 and 2025, and encompassed a range of study designs, including 9 randomized clinical trials, 17 prospective investigations, 23 retrospective studies, and 2 combining both retrospective and prospective analyses. The cohort sizes of the included studies ranged from 50 to 3138 patients, with a median of 238 (interquartile range (IQR) 124–467). The temporal distribution of publications is illustrated in Figure 3. Most studies originated from Italy (11), the USA (6), China (5), and Japan (5). Smaller numbers came from India, Canada, Germany, Brazil, South Korea, and several other countries in Europe, Asia, and South America.

## 4. Discussion

### 4.1. Characteristics of Included Studies

In this scoping review, we identified studies that quantitatively analyzed the size of PH after US-guided PRB. Only a subset of the available literature met the inclusion criteria, reflecting the fact that systematic reporting of PH size remains uncommon.

Remarkably, the temporal distribution of the included studies showed a progressive increase in publications over the last decade (Figure 3). This growing research interest may be linked to the observation, reported in several studies, that small and clinically insignificant PH occurs in the vast majority of patients after PRB [3,43,66,68]. As a result, reporting only the frequency of PH provides limited clinical insight.

### 4.2. Imaging Modalities

Among the 51 studies included in this review, 50 relied on US for PH assessment. This predominance reflects both its central role in clinical practice and its practicality as a safe, readily available, and non-invasive technique [69,70]. Beyond its classical use for detecting arteriovenous fistulas, some studies employed Doppler in the context of bleeding. Granata et al., 2011 [53] used it before puncture to avoid vascular structures, while Brabrand et al., 2013 [44] applied it systematically after each pass to monitor blood leakage, with five out of seven PH showing Doppler signal for more than 120 s. These applications provided additional insight into the dynamics of PH formation. Similarly, Pinto-Silva et al., 2025 [54] performed Doppler immediately after each puncture to detect active bleeding.

However, several limitations of US-based PH measurements should be acknowledged: PH often demonstrate irregular morphology and heterogeneous echogenicity [15,71,72,73,74] that evolves over time. Moreover, US is inherently operator- and equipment-dependent, and in obese or inadequately prepared patients, PHs may be difficult to visualize. [75] reflects the general limitation of US in assessing the retroperitoneal space [69]. This disadvantage is less relevant in transplanted kidneys, which are more superficially located [1]. Although reported in the renal transplant setting, US has tendency to underestimate PH size [76]. For instance, Fananapazir et al., 2015 [74] demonstrated that 27% of clinically significant postoperative PHs in renal graft recipients were not detected on US, and in 50% of cases, the PH volume was underestimated. Taken together, these factors indicate that US-based measurements of PH size may be prone to systematic variability and potential bias, which could partly explain discrepancies across studies and complicate interpretation.

Historically, computed tomography (CT) played a more prominent role in post-PRB PH assessment, particularly in the 1980s. A key conclusion from these studies was that PHs occur far more frequently than suggested by US-based reports, with detection rates reaching up to 90.9% [77]. However, most of these early CT studies did not meet our inclusion criteria, mainly due to small cohort sizes [78,79,80] or the absence of quantitative PH measurements [77]. As a result, only one CT-based study by Chikamatsu et al., 2017 [32] was included. We identified no other contemporary studies utilizing CT to quantify post-PRB PH size.

As of 27 August 2025, we did not find any published studies using other modalities; notably, no study quantitatively assessed post-PRB PH size with magnetic resonance imaging (MRI).

### 4.3. Perinephric Hematoma Measurement and Reporting

As pointed out by previous studies, there is no standard method of PH size measurement [6,32]. Across the included research, PH size was quantified using a spectrum of approaches. The predominant method was one-dimensional linear measurement (largest diameter or thickness), while two-dimensional surface-area methods were used less frequently and three-dimensional volumetry was used only in a minority (Table 2). This preference likely reflects the simplicity and feasibility of one-dimensional measurements within routine US workflows, whereas two-dimensional or three-dimensional techniques require more time and effort. On the other hand, PHs often have irregular morphology, making single-dimension measurements challenging and potentially unrepresentative of their true size—a limitation acknowledged by several authors in the literature [32]. The only CT-based study [32] uniquely applied volumetric segmentation. Notably, most studies did not provide an illustrative figure of the measurement technique, further limiting between-study comparability.

Imaging schedules varied considerably across studies. Some protocols included immediate US after PRB [3,18,24,25,30,31,36,41,43,47,52,54,58,60,61,64], while others performed scans shortly thereafter, within 5 min to 1 h [26,29,38]. Several studies scheduled US up to 10 h after PRB, applied either as the first assessment [2,22,33,37,63] or as a repeat examination [3,18,36,60]. Other protocols relied on US at 24 h (or ‘next morning’) [11,18,22,24,26,28,32,34,39,42,51,56,58,59,60] or 48 h [27,40,45,62], again either as the initial or as a subsequent follow-up assessment. In addition, a few reports extended the observation period further, including follow-up [3,29,30,35]. Some studies tailored their imaging protocols to clinical circumstances, with one study performing US only when clinically indicated [27,46], and another not specifying the exact timing of post-biopsy imaging [55].

The timing of post-PRB imaging has important implications, since complications do not always develop immediately after the procedure. Several authors have pointed out that a considerable proportion of complications can be detected within the first hours after biopsy, while others become evident only later, for example within 24 h. For instance, according to Simard-Meilleur et al., 2014 [68], 84% of complications manifested within the first 8 h, 86% within 12 h, and 94% within 24 h, while Whittier et al., 2004 [9] reported that major complications were apparent in only 67% of patients by 8 h but in more than 90% by 24 h. Thus, the chosen observation point directly affects the proportion of complications that are reported.

In addition to the variability in imaging timing, an important source of bias across the included studies is that in most included studies, US was performed systematically in all subjects, whereas in others, imaging was reserved only for individuals who developed clinical symptoms [34,37,46], while asymptomatic patients did not undergo routine assessment. Selective imaging inevitably results in under-detection of small or clinically silent PHs, which in turn lowers the reported incidence. In contrast, cohorts undergoing universal post-biopsy imaging demonstrate substantially higher PH frequencies [6]. This heterogeneity in imaging strategies further limits the interpretability of incidence data and highlights the need to focus on PH size and its clinical implications, which are less affected by whether PH are actively sought in every patient.

The reported frequency of PH occurrence shows a very wide range, from as low as 1.1% [64] to as high as 85% [43,66], highlighting the substantial heterogeneity in the literature. The CT-based study [32] selectively included subjects with PHs. This overall picture is consistent with the observations of other authors, who have likewise emphasized the marked variability between studies [6,68]. In our view, such variability mainly reflects differences in imaging protocols and timing, as well as the inherent limitations of US. Beyond these well-recognized factors, our review highlights an additional aspect not emphasized in earlier systematic reviews: several authors reported only PHs exceeding a predefined size threshold. For instance, Moledina et al., 2018 [30] reported only PHs > 5 cm, Tabatabai et al., 2009 [46] and Chen et al., 2012 [34] only PHs > 4 cm, Fontana et al., 2022 [59] only PHs > 3 cm, Manno et al., 2011 [24] and Hergesell et al., 1998 [25] restricted reporting to PHs ≥ 2 × 2 cm, and Sawicka et al., 2019 [31] and Jaturapisanukul et al., 2023 [23] considered only those > 1 cm. In contrast, Hogan et al., 2020 [65], Tanaka et al., 2017 [66] and Fisi et al., 2012 [28] reported every PH, even the tiniest PH, which is explicitly emphasized in their works. This is an additional factor that may help explain the considerable variation in PH frequencies observed between different authors.

There is also no uniform approach to reporting PH size. Some authors, especially those not accounting for the smallest PHs, reported only the incidence of cases above a certain cut-off (e.g., ≥1 cm, >3 cm or >5 cm) [23,27,35,55,59]. Others adopted a categorical approach, presenting the distribution of patients across predefined size ranges (e.g., ≤2 cm, 2–3 cm, >3 cm or >5 cm) [27,31,33,38]. Still, other studies reported central values. Among studies reporting a central one-dimensional metric, mean or median PH diameters ranged from approximately 13 mm to 30 mm, typically 20 to 30 mm. [28,42,44,56]. Among studies reporting two-dimensional measurements, most calculated surface area as the product of the longest and shortest diameters, with median or mean values typically ranging from ~200 to 400 mm^2^ and extending from as low as 0 mm^2^ up to 848 mm^2^ [11,24,49,51,66]. Among studies reporting volumetric data, PH sizes showed the widest variation. Chikamatsu et al., 2017 [32] reported a median of 38 mL (IQR 18–85 mL). Antunes et al., 2018 [3] described progressive increases in complicated cases, with mean volumes rising from 44 mL immediately post-PRB to 81 mL at day 7, compared to stable ~6–7 mL in uncomplicated cases. Constantin et al., 2010 [41] found mean PH volumes of 29 mL when using 16-gauge needles and 110 mL with 14-gauge needles. Meola et al., 1994 [26] noted that most PHs were very small, often <5 mL. Sattari et al., 2022 [4] reported mean PH volumes of 2.31 ± 1.17 mL, with desmopressin versus 7.72 ± 5.45 mL without it. Overall, reported central values therefore ranged from just a few milliliters up to 110 mL. In the case of outlying values, some studies reported them separately, often accompanied by varying levels of clinical detail [11,47,56].

### 4.4. Perinephric Hematoma Size and Location Significance

Despite these differences in methodology and reporting, there is general agreement across studies that the vast majority of PHs are small. However, no less variability concerns the very definitions of what constitutes a ‘small’ or ‘large’ PH, with thresholds ranging from 2 cm to 5 cm or based on area or volume criteria. Gesualdo et al., 2008 [29]. considered hematomas < 5 cm^2^ as minor complications. Some other authors used volumetric cut-offs, such as Chikamatsu et al., 2017 [32], who defined ‘massive bleeding’ as ≥85 mL on CT, or Antunes et al., 2018 [3], who considered volumes > 40 mL on US clinically significant. Independently, Xu et al., 2022 [62]. regarded a threshold of 40 mL as an indication for further diagnostic work-up, while volumes < 5 mL were considered negligible, not warranting additional evaluation. The specific definitions of ‘large’ PH applied in included studies are summarized in Table 3, further illustrating the lack of standardization and complicating direct comparisons.

Although many researchers reported that most post-PRB PHs are small and clinically silent, several groups have identified explicit thresholds beyond which PHs predict adverse outcomes. For example, Castoldi et al., 1994 [15] showed that PHs ≥ 2 cm were always symptomatic, and those ≥ 3 cm often caused severe complications, while Ishikawa 2009 [43] demonstrated that a width ≥ 2 cm on immediate ultrasound was the strongest predictor of Hb decline ≥ 10%. Similarly, Waldo et al., 2009 [38] found that PHs > 3 cm were associated with complicated courses, and Azmat et al., 2017 [52] reported that PHs ≥ 5 cm almost invariably required transfusion. Manno et al., 2004 [11] observed that, in general, larger PH surface areas were associated with major complications, although this was not always the case. Volume-based analyses also pointed to clinically relevant cut-offs: Chikamatsu et al., 2017 [32] linked PHs ≥ 85 mL with transfusion risk, and Bhattacharya et al., 2024 [18] showed that even relatively small early PHs (~1.2 cm within 12 h) accurately predicted subsequent transfusion. A comprehensive summary of thresholds and their clinical implications is provided in Table 4. On the other hand, some included studies did not demonstrate any significant difference in PH size between minor and major complications (e.g., Mejía-Vilet et al., 2018 [42], Fraser et al., 1995 [33], Helenius et al., 1983 [47]).

As far as biochemical endpoints are concerned, only a limited number of studies directly examined whether PH size related to post-PRB Hb decline. Among those that did, Ishikawa et al., 2009 [43] reported that hematomas ≥ 2 cm were the strongest predictor of an Hb drop ≥ 10%. Waldo et al., 2009 [38] and Eiro et al., 2005 [35] likewise noted greater reductions in Hb among patients with hematomas > 3 cm. Tanaka et al., 2017 [66] demonstrated a statistically significant linear correlation between PH size and next-day Hb decrease (r = 0.19, *p* < 0.0001). Murray et al., 2025 [55] found that patients with PHs ≥ 5 cm showed a more pronounced fall in Hb. These studies represent the subset of available data in which a size–Hb relationship was explicitly assessed.

Another aspect with potential clinical relevance is the location of the PH. Large PHs may exert a mass effect on the renal parenchyma and vascular pedicle or ureter [76]. Such compression can impair venous outflow or arterial inflow, thereby compromising renal perfusion and precipitating acute renal failure and anuria [74,76,81,82,83]. By contrast, subcapsular collections, confined beneath the relatively non-compliant renal capsule, increase intracapsular pressure and compress the underlying parenchyma. This pathophysiology underlies the so-called Page kidney phenomenon, in which persistent parenchymal compression activates the renin–angiotensin–aldosterone system and leads to secondary hypertension [84,85,86].

As noted in Section 2.5, most studies used overlapping terms and rarely localized PH precisely, making location-specific inferences difficult. Only 9/51 (17.7%) of included reports recorded location systematically—for example, Rapaccini et al., 1983 [40] (subcapsular vs. perirenal), Fisi et al., 2012 [28] (perirenal vs. intrarenal vs. retroperitoneal), Pirklbauer et al., 2022 [39] (perinephric vs. subcapsular in allografts), Zhang et al., 2019 [67]. (perinephric/suprarenal/subcapsular), Meola et al., 1994 [26] (perirenal/pararenal), and Tabatabai et al., 2009 [46] (retroperitoneal/perinephric)—underscoring how uncommon detailed localization was among included studies. To improve clarity, we therefore documented in Supplementary Table S2 how the included studies reported PH location.

In our review, all terms were grouped under the overarching concept of PH, reflecting the inconsistent and overlapping terminology across studies. While this approach allowed for a more comprehensive synthesis, it inevitably limited the ability to distinguish potential differences in clinical significance between subcapsular, perinephric, and retroperitoneal PH.

In daily practice, however, post-PRB monitoring is still guided primarily by patient observation and laboratory values rather than systematic imaging. This approach reflects the fact that most PHs are small and clinically silent. Nonetheless, the evidence summarized above suggests that PH size and location may provide additional prognostic information, and its more consistent assessment and reporting could improve risk stratification in selected patients.

### 4.5. Factors Influencing Perinephric Hematoma Size

While some authors did not identify any determinants of PH size [11], others have reported several factors associated with its variation. Some authors indicated that technical aspects of the procedure influenced PH size. They found that the use of smaller PRB needles (18 G) reduces the risk of large PHs compared with 16 G or older 14 G needles, while still providing adequate tissue yield [3,49,56,62]. Others pointed out that the number of passes was also important: performing ≥ 4 passes markedly increased the likelihood of large PHs [32,55]. Needle trajectory also mattered: An oblique puncture at 50–70° directed to the poles while avoiding the medulla lowered the frequency of PHs > 5 cm and transfusions [31,55], and a caudal approach resulted in fewer PHs ≥ 1 cm and fewer transfusions compared with a cranial approach [23]. In addition, institutional protocols—including checklists, limiting the number of operators, on-site microscopy, and consistent ultrasound guidance—have been shown to halve the rate of large PHs [55].

The effect of pharmacological prophylaxis is less consistent. Some trials reported that desmopressin reduced the incidence and size of PHs [4,24], yet more recent data suggest that it does not necessarily reduce PH volume but rather accelerates resorption, resulting in a lower prevalence at 24 h [22]. By contrast, tranexamic acid has not proven beneficial; in fact, a randomized controlled trial reported that high dose of tranexamic acid was associated with larger PHs compared with placebo [51], providing no support for its routine use in this setting.

Clinical characteristics of the patient were also significant determinants. Impaired kidney function, (low estimated glomerular filtration rate (eGFR), high blood urea nitrogen (BUN)), hospitalization, acute kidney injury, and dialysis all predisposed a patient to large PHs and transfusion [30,37,42,50]. Anemia, thrombocytopenia, and prolonged coagulation times (APTT, PT and INR) came out as predictors of major bleeding events [34,42,46]. Other risk factors included female sex [11,30], elevated periprocedural blood pressure [37,43], and younger age or lower body mass index (BMI) [28], whereas older age and obesity have sometimes been reported as protective [57]. Histopathologic context also matters, with vasculitis and lupus nephritis carrying higher bleeding risk than diabetic nephropathy or acute tubular necrosis [28]. It should be noted that the use of low-dose aspirin did not increase the risk of large PHs in a large contemporary cohort [59].

### 4.6. Future Research

In terms of future research directions, several avenues can be envisioned. Contrast-enhanced ultrasound (CEUS) could improve detection and quantification of post-PRB PHs, as the technique enhances the conspicuity of blood collections and may reduce the variability inherent to conventional US-based measurements. CEUS has already been applied for identification of post-operative PHs, including the transplant kidney setting [69,72,76,87]. Moreover, CEUS has been shown to detect active bleeding in hematomas (e.g., via jet-like enhancement in soft-tissue hematomas) [88,89,90]. We identified one original study employing CEUS [91]; however, it was only available as an abstract (full text in Chinese), which precluded detailed analysis.

CT is generally considered the most appropriate modality to evaluate retroperitoneal bleeding [69,92], particularly for the confirmation and delineation of PHs detected on US and for procedural planning, although it is associated with inherent disadvantages such as exposure to ionizing radiation and the need for intravenous iodinated contrast media administration [69], which may be a limitation in the context of renal diseases. It should be acknowledged that CT has the capability to detect active bleeding when intravenous contrast is administered [69,92]; however, there are also reports describing its potential to suggest ongoing hemorrhage even on non-contrast studies [92]. Nonetheless, CT role has become less prominent in research on post-PRB complications in recent years.

MRI represents another promising modality: thanks to its high soft-tissue resolution [69] and ability to assess the age of blood products (acute/subacute vs. chronic) [69], MRI could provide more precise characterization of PHs, although its use will likely remain confined to research settings due to cost and limited practicality (e.g., long examination time) for routine clinical workflows [69].

Beyond new imaging modalities, progress may also come from advances in image analysis and segmentation techniques. Automated or semi-automated segmentation has the potential to provide precise and reproducible volumetric measurements of post-PRB PHs. Such approaches could overcome the subjectivity and inter-observer variability inherent to manual measurements, especially in cross-sectional modalities like CT or MRI. Moreover, with the rapid development of artificial intelligence–based methods, it is conceivable that PH size could be quantified fully automatically, enabling large-scale, standardized data collection in both clinical practice and research. This, in turn, would facilitate more accurate risk stratification and improve comparability across studies.

Future studies should prioritize standardized definitions, unified measurement techniques, and consistent reporting practices. Only through consistent definitions, unified measurement techniques, and systematic reporting will it be possible to generate evidence that can be meaningfully compared across cohorts. Such an approach is essential to translate heterogeneous research findings into robust clinical guidance and to establish the true prognostic value of PH size after PRB.

## 5. Limitations

This scoping review has several limitations. First, as a literature-based study, it relied entirely on published data and may therefore be subject to reporting bias. Only articles written in English were included, which could have resulted in the exclusion of relevant evidence available in other languages. Grey literature was not systematically searched, raising the possibility of publication bias. Furthermore, no formal appraisal of methodological quality or risk of bias of the included studies was performed. However, this is consistent with the nature of a scoping review, which is intended to map the available evidence and identify knowledge gaps rather than to provide graded evidence for clinical recommendations. Finally, the included studies themselves were highly heterogeneous in terms of design, imaging protocols, and outcome reporting, which limited the comparability of results across studies.

## 6. Conclusions

This scoping review highlights that research on post-PRB PHs has traditionally focused on incidence, yet the available evidence indicates that PH size carries additional clinical significance. While most PHs are small and clinically silent, several studies identified thresholds above which the risk of adverse outcomes increases substantially. These findings suggest that systematic assessment and reporting of PH size, beyond incidence alone, provide important prognostic insights. Therefore, future studies should aim to establish a consensus on post-biopsy imaging assessment, define the preferred imaging modality, standardize the approach to quantifying PH presence and size, and optimize timing for planned post-PRB evaluation. Such consensus would facilitate comparability across studies and clarify the true clinical value of PH size after PRB.

## Figures and Tables

**Table 1:** Studies included in scoping review.

Study Design	Imaging Modality	Authors (Year, Country)
RCT	US	Kim et al. (1998, Republic of Korea) [49]; Gesualdo et al. (2008, Italy) [29]; Manno et al. (2011, Italy) [24]; Antunes et al. (2018, Brazil) [3]; Pokhrel et al. (2018, Nepal) [63]; Sattari et al. (2022, Iran) [4]; Izawa et al. (2023, Japan) [51]; Jaturapisanukul et al. (2023, Thailand) [23]; Chakrabarti et al. (2025, India) [22]
Prospective	US	Helenius et al. (1983, Finland) [47]; Rapaccini et al. (1989, Italy) [40]; Meola et al. (1994, Italy) [26]; Castoldi et al. (1994, Italy) [15]; Fraser et al. (1995, Australia) [33]; Manno et al. (2004, Italy) [11]; Eiro et al. (2005, Japan) [35]; Schwarz et al. (2005, Germany) [48]; Maya et al. (2009, USA) [36]; Waldo et al. (2009, USA) [38]; Brabrand et al. (2012, Norway) [44]; Tanaka et al. (2017, Japan) [66]; Moledina et al. (2018, USA) [30]; Zhang et al. (2019, China) [67]; Hogan et al. (2020, USA) [65]; Asad et al. (2021, India) [50]; Bhattacharya et al. (2024, India) [18]
Mixed (retrospective + prospective cohort)	US	Wang et al. (2015, China) [37]; Mejía-Vilet et al. (2018, Mexico) [42]
Retrospective	US	Hergesell et al. (1998, Germany) [25]; Boschiero et al. (1992, Italy) [45]; Ishikawa et al. (2009, Japan) [43] Tabatabai et al. (2009, USA) [46]; Constantin et al. (2010, Canada) [41]; Granata et al. (2011, Italy) [17]; Chen et al. (2012, USA) [34]; Fisi et al. (2012, Hungary) [28]; Lubomirova et al. (2014, Bulgaria) [56]; Azmat et al. (2017, Pakistan) [52]; Brardi et al. (2018, Italy) [61]; Sawicka et al. (2019, Canada) [31]; Fontana et al. (2022, Italy) [59]; Garozzo et al. (2022, Italy) [60]; Xu et al. (2022, China) [62]; Pirklbauer et al. (2022, Austria) [39]; Li et al. (2024, China) [27]; Demirelli et al. (2024, Turkey) [58]; Jung et al. (2025, Republic of Korea) [57]; Pinto-Silva et al. (2025, Brazil) [54]; Murray et al. (2025, Canada) [55], Tsai et al. (2016, Taiwan) [64]
Retrospective	CT	Chikamatsu et al. (2017, Japan) [32]

RCT—randomized clinical trial; US—ultrasound; CT—computed tomography.

**Table 2:** Methods of perinephric hematoma measurement across included studies.

Measurement Approach	Definition (Typical)	Number of Studies (n = 51)
One-dimensional	Largest diameter or thickness	31
Two-dimensional	Product of two diameters (diameter × diameter or surface area)	12
Three-dimensional (volume)	Ellipsoid formula or modified ellipsoid formula	7
Three-dimensional (volume)	Image segmentation	1

**Table 3:** Definitions of large perinephric hematomas in the included studies.

Measurement Method	Threshold	Authors
One dimension (single maximal diameter/thickness/depth)	≥2 cm	Ishikawa et al., 2009 [43]; Asad et al., 2001 [50]
	>3 cm	Eiro et al., 2005 [35]; Schwarz et al., 2005 [48].; Waldo et al., 2009 [38]; Wang et al., 2015 [37]; Castoldi et al.1994 [15]; Fraser et al., 1995 [33]
	≥4 cm	Chen et al., 2012 [34]; Granata et al., 2011 [17]
	≥5 cm	Moledina et al., 2018 [30]; Azmat et al., 2017 [52]; Sawicka et al., 2019 [31]; Hogan et al., 2020 [65]; Li et al., 2024 [27]; Murray et al., 2025 [55]; Jung et al., 2025 [57]; Xu et al., 2022 [62]; Bhattacharya 2024 [18]
Two dimensions (dimension × dimension)	> 3 × 1 cm	Castoldi et al., 1994 [15]
Two dimensions (surface area)	≥464.0 mm^2^	Manno et al., 2004 [11]
Three dimensions (volume)	≥40 mL (US)	Xu et al., 2022 [62]
	≥40 mL (US)	Antunes et al., 2018 [3]
	≥50 mL (US)	Pinto-Silva et al., 2025 [54]
	≥85 mL (CT)	Chikamatsu et al., 2017 [32]
	≥100 mL (US)	Meola et al., 1994 [26]

US—ultrasound; CT—computed tomography.

**Table 4:** Clinical significance thresholds for perinephric hematoma size in the included studies.

Authors	Threshold Definition	Clinical Consequences
Castoldi et al., 1994 [15]	Thickness ≥ 2 cm; ≥ 3 cm	≥2 cm: all symptomatic; ≥3 cm: severe complications in 6/7 (86%)
Ishikawa et al., 2009 [43]	Width ≥ 2 cm (immediate US)	Strongest predictor of Hb drop ≥ 10% (OR 8.07); mean Hb fall 6.9% vs. 2–3% if <2 cm
Waldo et al., 2009 [38]	Diameter > 3 cm at 1 h	In complicated cases, 55% > 3 cm vs. 26% in uncomplicated; absence of ‘large’ PH = NPV 95–98% for safe course
Wang et al., 2015 [37]	Depth > 3 cm	‘Large’ PH linked to major complications; targeted interventions reduced incidence (3.6 → 0.2%)
Azmat et al., 2017 [52]	Diameter ≥ 5 cm	87% of ≥5 cm PH required transfusion; overall transfusion rate 7.4%
Moledina et al., 2018 [30]	≥5 cm or “moderate/large”	PH ≥ 5 cm in 7% of subjects; associated with transfusion (8%) and angiographic intervention (2%)
Hogan et al., 2020 [65]	PH > 5 cm	4% of cases; linked with transfusion (2%), gross hematuria (2%), prolonged hospitalization (4%)
Manno et al., 2004 [11]	Area surface of 464.0 mm^2^	Median area surface in subgroup with major complications
Chikamatsu et al., 2017 [32]	CT volume ≥ 85 mL (“massive”)	4.7% transfusion; ≥85 mL defined upper tertile of bleeding
Antunes et al., 2018 [3]	Hematoma volume (serial US)	Complicated cases: volume rose from 44 → 81 mL over days 0–7; uncomplicated: stable ~6–7 mL
Bhattacharya et al., 2024 [18]	Diameter ≥ 1.17 cm (0 h) or ≥1.2 cm (12 h)	Predicted transfusion with 100% sensitivity, ~70% specificity; ‘large’ PH > 5 cm in 6%
Pinto-Silva et al., 2025 [54]	US volume > 50 mL	PHs > 50 mL referred to emergency department

PH—perinephric hematoma; Hb—hemoglobin; OR—odds ratio; NPV—negative predictive value; CT—computed tomography; US—ultrasound.

## Data Availability

No new datasets were generated or analyzed during the current study. All data used in this scoping review are derived from previously published articles cited in the manuscript.

## Supplementary Materials

The following supporting information can be downloaded at: https://www.mdpi.com/article/10.3390/biomedicines13122943/s1. Table S1. Full database search strategies used in the scoping review. Table S2. Characteristics of studies included in scoping review.
