# Supplementary material for: Reporting of Perirenal Hematoma Size After Ultrasound-Guided Renal Biopsy in Adults: A Scoping Review
Source: Biomedicines. 2025 Nov 29;13(12):2943. doi: 10.3390/biomedicines13122943 (PMC12730393; doi:10.3390/biomedicines13122943)
Supplement: Supplementary file 1 [file biomedicines-13-02943-s001.zip › Supplementary Table S1.pdf]

Supplementary Table S1. Full database search strategies used in the scoping review

Database	Search strategy
PubMed/MEDLINE	("Biopsy/adverse effects"[MeSH] OR "Biopsy, Needle/adverse effects"[MeSH] OR "Image-Guided Biopsy/adverse effects"[MeSH] OR "Biopsy/methods"[MeSH] OR "Biopsy, Needle/methods"[MeSH] OR "Image-Guided Biopsy/methods"[MeSH] OR renal biopsy[tiab] OR kidney biopsy[tiab] OR percutaneous biopsy[tiab] OR percutaneous kidney biopsy[tiab] OR percutaneous renal biopsy[tiab]) AND ("Kidney/pathology"[MeSH] OR "Kidney/diagnostic imaging"[MeSH] OR "Kidney Diseases/pathology"[MeSH] OR "Kidney Diseases/diagnostic imaging"[MeSH] OR "Kidney Diseases/etiology"[MeSH] OR "Kidney Transplantation"[MeSH] OR renal[tiab] OR kidney[tiab]) AND ("Hematoma/etiology"[MeSH] OR "Hematoma/diagnostic imaging"[MeSH] OR "Hemorrhage/etiology"[MeSH] OR "Hemorrhage/diagnostic imaging"[MeSH] OR hematoma*[tiab] OR hemorrhag*[tiab] OR bleeding[tiab] OR perirenal hematoma[tiab] OR perinephric hematoma[tiab] OR complication*[tiab]) AND ("Ultrasonography"[MeSH] OR "Ultrasonography, Interventional"[MeSH] OR "Tomography, X-Ray Computed"[MeSH] OR "Magnetic Resonance Imaging"[MeSH] OR ultrasound[tiab] OR ultrasonograph*[tiab] OR CT[tiab] OR "computed tomography"[tiab] OR MRI[tiab] OR "magnetic resonance imaging"[tiab] OR imaging[tiab])
Embase	('kidney biopsy'/exp OR "renal biopsy":ti,ab OR "kidney biopsy":ti,ab OR "percutaneous renal biopsy":ti,ab OR "percutaneous kidney biopsy":ti,ab) AND ("perirenal hematoma":ti,ab OR "perinephric hematoma":ti,ab OR hematoma*:ti,ab OR hemorrhag*:ti,ab OR bleeding:ti,ab) AND ('ultrasonography'/exp OR 'computed tomography'/exp OR 'magnetic resonance imaging'/exp OR ultrasound:ti,ab OR ultrasonograph*:ti,ab OR CT:ti,ab OR "computed tomography":ti,ab OR MRI:ti,ab OR "magnetic resonance imaging":ti,ab)
Cochrane [CENTRAL]	**Keywords used:** renal biopsy; kidney biopsy; percutaneous renal biopsy; percutaneous kidney biopsy; hematoma; hematomas; hemorrhage; bleeding; perirenal hematoma; perinephric hematoma; ultrasound; ultrasonography; computed tomography; CT; magnetic resonance imaging; MRI
Scopus	TITLE-ABS-KEY("renal biopsy" OR "kidney biopsy" OR "percutaneous renal biopsy" OR "percutaneous kidney biopsy")

Database	Search strategy
	AND TITLE-ABS-KEY(hematoma* OR hemorrhag* OR bleeding OR "perirenal hematoma" OR "perinephric hematoma" OR complication*) AND TITLE-ABS-KEY(ultrasound OR ultrasonograph* OR "computed tomography" OR "CT" OR "MRI" OR "magnetic resonance imaging")

CENTRAL -Central Register of Controlled Trials
