# Supplementary material for: Reporting of Perirenal Hematoma Size After Ultrasound-Guided Renal Biopsy in Adults: A Scoping Review
Source: Biomedicines. 2025 Nov 29;13(12):2943. doi: 10.3390/biomedicines13122943 (PMC12730393; doi:10.3390/biomedicines13122943)
Supplement: Supplementary file 1 [file biomedicines-13-02943-s001.zip › Supplementary Table S2.pdf]

Supplementary Table S2. Characteristics of studies included in scoping review

Author (Year)	Imaging modality	Study population (country, number of subjects (% male), mean age, native vs transplanted kidneys)	Measurement method (1D/2D/3D)	Hematoma size cutoff	Clinical outcomes associated with hematoma size	Reporting hematoma location (systematic classification - YES/NO; terms used)
**Kim et al. (1998) [49]**	US	South Korea n = 166 (54% male) mean age 35.5 ± 15.6 years native kidneys only	2D (surface area = longest × shortest diameter)	None defined	Manual 14G biopsy caused larger and more frequent hematomas than automated biopsy	NO<br><br>All reported as perirenal
**Gesualdo et al. (2008) [29]**	US	Italy n = 110 (57% male); mean age 45.6 ± 12.8 years; native kidneys only	2D (surface area = longest × shortest diameter)	Minor hematoma <5 cm²	Minor complications 7.6% (perinephric hematoma <5 cm², pain, hematuria); major complications 0.9% (transfusion/angiography); no nephrectomy, no deaths	NO<br><br>All reported as subcapsular perinephric
**Manno et al. (2011) [24]**	US	Italy n = 162 (54% male); mean age 40.6 ± 14.6 years; native kidneys only	2D (diameter x diameter	Hematomas <20×20 mm NR at all	DDAVP reduced hematoma size (13.7% vs 30.5%); no transfusion, AVF formation, or intervention.	NO<br><br>All reported as perirenal
**Antunes et al. (2018) [3]**	US	Brazil n = 238 (51.7% male); mean age 40.2 ± 15.7 years; 82% native kidneys / 18% transplant	3D (volumetric; ellipsoid formula)	≥40 mL predictive for complications	Larger hematomas in subjects with complications: 44 ± 56 mL vs 5.9 ± 6.6 mL	NO<br><br>All reported as perirenal

**Pokhrel et al. (2018) [63]**	US	Nepal<br>n = 75 (52% male);<br>mean age 33.5 ± 15.5 years;<br>native kidneys only	1D (exact measurement method not specified)	None defined	No hematomas>2 cm; all hematomas formed ≤6 h no size–outcome analysis	NO<br><br>All reported as perinephric
**Sattari et al. (2022) [4]**	US	Iran<br>n = 120 (48% male);<br>mean age 45.3 ± 16.0 years;<br>native kidneys only	3D (volumetric; length × width × depth)	None defined	Lower hematoma incidence and smaller volumes with DDAVP; no major complications	NO<br><br>All reported as perirenal
**Izawa et al. (2023) [51]**	US	Japan<br>n = 56 (52% male);<br>median age 56 years (IQR 46–70); native kidneys only	2D (surface area; exact measurement method not specified)	None defined	Hematoma area larger in high-dose TXA vs placebo	NO<br><br>All reported as perirenal
**Jaturapisanukul et al. (2023) [23]**	US	Thailand<br>n = 107 (38% male);<br>mean age 41.6 ± 13.3 years; native kidneys only	1D (maximum diameter)	Hematomas < 1 cm NR at all	No difference in hematomas incidence or size between cranial vs caudal group	NO<br><br>All reported as perinephric
**Chakrabarti et al. (2025) [22]**	US	India<br>n = 152 (51% male);<br>mean age 39 ± 15 years;<br>native kidneys only	3D (volumetric; exact measurement method not specified)	None defined	Hematoma volumes small in both groups (median 1–3 mL at 6 h and 24 h); no difference between desmopressin vs placebo; transfusion 0%; embolization 0%; no surgery, no deaths	NO<br><br>All reported as perinephric
**Helenius et al. (1983) [47]**	US	Finland<br>n = 57 (50% male);<br>mean age 37 years	1D (maximum thickness)	None defined	Severe flank pain in large hematomas (4 cm × 5×10 cm); most	NO

		(range 18–74); native kidneys only			hematomas clinically mild; no size–outcome analysis	All reported as perirenal
**Rapaccini et al. (1989) [40]**	US	Italy<br>n = 114 (50% male); mean age 37.8 years (range 8–82); native kidneys only	1D (maximum diameter)	None defined	All hematomas small (2–3 cm), clinically asymptomatic; resolved spontaneously	YES<br><br>• Subcapsular<br>• Perirenal
**Meola et al. (1994) [26]**	US	Italy<br>n = 596 (47% male); mean age NR (range 11–88); native kidneys only	3D (volumetric; ellipsoid formula)	• Blood spilling <5 ml<br>• Large hematoma >100 mL	<5 ml spills asymptomatic; >100 ml hematoma symptomatic (drop in RBC, transfusion)	YES<br><br>• Perirenal<br>• Pararenal
**Castoldi et al. (1994) [15]**	US	Italy<br>n = 230 (sex NR); age NR; native kidneys only	2D (thickness and length)	• Small: thickness < 1 cm and length < 3 cm<br>• Medium: thickness < 1 cm and length ≥ 3 cm<br>• Large: thickness ≥ 1 cm and length ≥ 3 cm	>2 cm symptomatic; >3 cm severe complications; large hematomas linked with Hb drop & intervention.	YES<br><br>• Parenchymal + perirenal<br>• Subcapsular<br>• Perirenal<br>• Subcapsular + perirenal<br>• Undefinable
**Fraser et al. (1995) [33]**	US	Australia<br>n = 336 (33% male); median age 39 years (IQR NR); native kidneys only	1D (maximum diameter)	Hematomas ≤3 cm clinically silent	Hematoma size correlated with symptoms — all ≤3 cm were clinically silent; symptomatic cases occurred only with larger hematomas	NO<br><br>All reported as perirenal

**Manno et al. (2004) [11]**	US	Italy<br>n = 471 (59% male); mean age 38.5 ± 15.3 years;<br>native kidneys only	2D (surface area = longest × shortest diameter)	464.0 mm2 = median area surface in subgroup with major complications	33.3% with hematoma<br>1.2% with major complications;<br>size not linked to predictors;<br>no size–outcome analysis	NO<br><br>All reported as subcapsular perinephric hematoma
**Eiro et al. (2005) [35]**	US	Japan<br>n = 359 (52% male); mean age 44.0 ± 17.2 years; native kidneys only	1D (thickness)	• Small ≤2 cm;<br>• Medium 2–3 cm;<br>• Large ≥3 cm	Thickness correlates with Hb drop; no severe cases	NO<br><br>All reported as perirenal
**Schwarz et al. (2005) [48]**	US	Germany<br>n = 508 (sex NR); age NR; transplanted kidneys only	1D (maximum diameter)	>3 cm associated with need for observation/hospitalization	Most hematomas small and clinically mild; 4/41 required transfusion	NO<br><br>All reported as perirenal
**Maya et al. (2009) [36]**	US	USA<br>n = 100 (40% male); mean age 41.9 ± 15.4 years;<br>native kidneys only	2D (two linear dimensions: height × width)	Small <2×2 cm	All hematomas <2×2 cm; no large hematomas; all clinically mild	NO<br><br>All reported as perinephric
**Waldo et al. (2009) [38]**	US	USA<br>n = 162 (38% male); mean age 47 ± 17 years; native kidneys only	1D (largest dimension)	• Small ≤1 cm;<br>• Medium 1–3 cm;<br>• Large ≥3 cm	>3 cm at 1 h associated with complications; ≤3 cm clinically mild; no hematoma at 1 h → 95% NPV for uncomplicated course	NO<br><br>All reported as perirenal
**Brabrand et al. (2012) [44]**	US	Norway<br>n = 71 (65% male); mean age 51 years; transplanted kidneys only	1D (diameter)	None defined	CD bleeding >120 s measured immediately after each needle pass strongly associated	NO<br><br>All reported as perirenal

					with hematoma formation; all hematomas ≤3.5 cm; all clinically minor	
**Tanaka et al. (2017) [66]**	US	Japan n = 462 (53% male); mean age 50.4 ± 18.2 years; native kidneys only	2D (surface area = longest × shortest diameter)	None defined	Median hematomas 1.5 cm² (IQR 0.99–2.2); nearly all hematomas minor; Hb drop correlates with hematoma area; ≥10% Hb drop in 11.7%	NO<br><br>All reported as perirenal
**Moledina et al. (2018) [30]**	US	USA n = 159 (43% male); median age 59 years (IQR 47–68); native kidneys only	1D (any dimension)	<5 cm NR at all	11/159 hematomas; median time to transfusion 13 h; no biopsy-related deaths; delayed hematomas identified only on symptom-triggered re-imaging	NO<br><br>Location NR at all
**Zhang et al. (2019) [67]**	US	China n = 122 (57% male); mean age 36.8 ± 12.9 years; native kidneys only	1D (exact measurement method not specified)	None defined;	All hematomas <5 cm Symptoms in 2/24; no transfusion; no intervention; no size–outcome analysis	YES<br><br>• Perinephric<br>• Subcapsular<br>• Suprarenal
**Hogan et al. (2020) [65]**	US	USA n = 160 (43% male); mean age 54 ± 13 years; native kidneys only	1D (exact measurement method not specified)	'Serious' >5 cm	'Serious' hematoma in 4.4%; 3 transfusions; no interventions; overall 7% complications	NO<br><br>Location NR at all
**Asad et al. (2021) [50]**	US	India n = 126 (75% male); mean age 40 ± 13	1D (longest diameter)	Large ≥2 cm	Hematomas in 37.3%; large hematomas ≥2 cm in 22.2%; major complications in 5.6%;	NO<br><br>All reported as perirenal

		years;<br>native kidneys only			most complications detected within 12 h; ≥2 cm hematomas more frequent when eGFR < 15	
**Bhattacharya et al. (2024) [18]**	US	India<br>n = 126 (75% male); mean age 40 ± 13 years;<br>native kidneys only	1D (longest diameter)	• >5 cm defined as major complication<br>• 1.2 cm at 12 h = 100% sensitivity for transfusion	41.3% hematomas; 6 major (>5 cm); 1.2 cm at 12 h = 100% sensitive predictor of transfusion; 98.9% detected ≤12 h.	NO<br>• Perinephric<br>• Subcapsular perinephric
**Wang et al. (2015) [37]**	US	China<br>n = 1314 (61% male); mean age 40 ± 15 years;<br>native kidneys only	1D (exact measurement method not specified)	Large >3 cm	Large hematomas (>3 cm) associated with major hemorrhagic complications; major complications 4.8% (retrospective) and 2.8% (prospective)	NO<br>Location NR at all
**Mejía-Vilet et al. (2018) [42]**	US	Mexico<br>n = 1205 (31% male); median age 33 years (IQR 25–47);<br>native kidneys only	1D (largest diameter)	None defined	6.8% major complications; hematomas more frequent in complicated cases; median largest diameter 30 mm vs 24 mm; hematoma size not predictive	NO<br>• Perinephric<br>• Perirenal
**Hergesell et al. (1998) [25]**	US	Germany<br>n = 1090 (63% male); median age ~47 years; 90% native / 10% transplanted	2D (dimension x dimension)	<2×2 cm NR at all	Hematomas (>2×2 cm) in 2.2%;<br>0.36% transfusions; no deaths; overall complication rate very low	NO<br>All reported as renal

**Boschiero et al. (1992) [45]**	US	Italy<br>n = 103 (63% male); mean age 39 years (SD NR); transplanted kidneys only	2D (dimension x dimension; not described in methods section)	None defined	5.3% perirenal blood collections (all small; largest ~3×1 cm); macroscopic hematuria 8.8%; AV fistulae 0.9%; no major hemorrhagic complications reported	NO<br><br>All reported as perirenal blood collections
**Ishikawa et al. (2009) [43**	US	Japan<br>n = 317 (54% male); mean age 45 ± 18 years;<br>native kidneys only	1D (maximum width)	• Small <2 cm<br>• Large ≥2 cm	Perirenal hematoma in 86%; 9.7% had hematomas ≥2 cm—the subgroup most strongly associated with Hb drop; overt Hb drop ≥10% occurred in 10.1%; major bleeding requiring intervention/transfusi on in 2.2%	NO<br><br>All reported as perirenal
**Tabatabai et al. (2009) [46]**	US	USA<br>n = 1024 (44% male); mean age 45 ± 15 years;<br>native kidneys only	1D (exact measurement method not specified)	• Minor complication ≥4 cm<br>• <4 cm NR at all	4.3% minor complications; major complications 1.2%; transfusion 0.6%; no biopsy-related deaths	YES<br><br>• Perinephric<br>• Retroperitone al
**Constantin et al. (2010) [41]**	US	Canada<br>n = 121 (≈53% male); mean age 52± 17 years ;<br>native kidneys only	3D (volumetric; modified ellipsoid formula = length × width × height × 0.5)	None defined	Asymptomatic perirenal hematoma in 27% (end-cut) and 25% (side-notch); mean volume 29 mL vs 110 mL; 1.5% vs 7.3% major bleeding; embolization required only in side-notch group	NO<br><br>All reported as perirenal

**Granata et al. (2011) [17]**	US	Italy<br>n = 561 (57% male); median age 46 years (IQR NR);<br>native kidneys only	1D (greater diameter in cm)	• Minor hematoma <4 cm<br>• Major hematoma>4 cm	Hematoma <4 cm in 3–8%; hematoma >4 cm or transfusion/interventi on in 0.4–2.1%.	NO<br><br>• Perirenal<br>• Subcapsular perinephric
**Chen et al. (2012) [34]**	US	USA<br>n = 219 (15% male); mean age 36.6 ± 13.3 years;<br>native kidneys only	1D (exact measurement method not specified)	• Minor complication ≥4 cm<br>• <4 cm NR at all	Minor complications in 7.8% ; major in 2.7% (intervention); no deaths	NO<br><br>All reported as perinephric
**Fisi et al. (2012) [28]**	US	Hungary<br>n = 353 (58% male); mean age 49 ± 14 years; native kidneys only	1D (details not specified)	None defined	Perirenal 40.5%; intrarenal 3.7%; retroperitoneal 1.1%; Major comlications in 2.8% (transfusion/emboliza tion/nephrectomy); no deaths	YES<br><br>• Perirenal<br>• Intrarenal<br>• Retroperitone al
**Lubomirova et al. (2014) [56]**	US	Bulgaria<br>n = 230 (52% male); mean age 45.5 ± 22.3 years;<br>native kidneys only	1D (exact measurement method not specified)	None defined	Hematomas in 26%; most ~20 mm);<br>3 larger (10–12 cm) with clinical bleeding and occasional transfusion; no embolization or deaths	YES<br><br>• Intrarenal<br>• Perirenal<br>• Intrarenal+pe rirenal
**Azmat et al. (2017) [52]**	US	Pakistan<br>n = 220 (37% male); mean age 41.6 ± 8.6 years;<br>native kidneys only	1D (exact measurement method not specified)	• Minor hematoma <5 cm<br>• Major hematoma >5 cm	Hematoma in 7.6%; among hematomas >5 cm, 87.5% required transfusion; no deaths	NO<br><br>• Perinephric<br>• Subcapsular perinephric

**Brardi et al. (2018) [61]**	US	Italy<br>n = 50 (64% male); mean age 52.7 ± 16.7 years;<br>native kidneys only	1D (thickness)	Non-significant hematoma ≤2 cm	40% hematomas (all ≤2 cm); 1 transient gross hematuria; no transfusions; no interventions	NO<br><br>All reported as subcapsular
**Sawicka et al. (2019) [31]**	US	Canada<br>n = 242 (56% male); median age 49 years (IQR 34–62);<br>81% native / 19% transplanted	1D (diameter)	• Large hematoma > 5 cm<br>• < 1cm NR at all	Bleeds >1 cm: 29%; bleeds >5 cm: 6%; no embolization/surgery; no deaths	NO<br><br>Location NR at all
**Fontana et al. (2022) [59]**	US	Italy<br>n = 722 (59% male); mean age 52.2 ± 17.7 years;<br>native kidneys only	1D (maximal diameter)	< 3 cm NR at all	Major 2.7% (transfusion/embolization/surgery/nephrectomy); no deaths	NO<br><br>Location NR at all
**Garozzo et al. (2022) [60]**	US	Italy<br>n = 322 (59% male); mean age 50.7 ± 15.7 (men) and 44.2 ± 16.4 (women); native kidneys only	1D (diameter)	Minor hematoma <5 cm	Minor: hematoma <5 cm (36%); AVF 2%; 0.51% abscess. Major: 2.85% transfusion; 0.3% embolization; no nephrectomy; no deaths	NO<br><br>All reported as subcapsular perinephric
**Xu et al. (2022) [62]**	US	China<br>n = 3138 (45% male); mean age 41.98 ± 14.17 years (18G) and 40.44 ± 14.29 years (16G);<br>native kidneys only	• 3D (volumetric; modified ellipsoid formula = length × width × height × 1/3)<br>• Additionally, 1D (long diameter)	• Small <50 mm<br>• Large ≥50 mm<br>• Regular monitoring > 40 mL<br>• Monitoring discontinued <5 mL	Large hematoma 6.6%; AVF 0.4%; transfusion ≤0.5%; 1 embolization; no deaths	NO<br><br>All reported as perirenal

**Pirklbauer et al. (2022) [39]**	US	Austria<br>n = 250 (62% male); median age 50 years (range 18–78); transplanted kidneys only	2D (length × thickness)	• Small <3x1 cm<br>• Large ≥3x1 cm	Major: transfusion 0.3%, embolization 0.1%, catheter 0.3%; no surgery, no graft loss, no deaths. Minor: perinephric 11.6%, subcapsular 1.0%, AVF 2.5%, gross hematuria 1.4%	YES<br>• Perinephric<br>• Subcapsular
**Li et al. (2024) [27]**	US	China<br>n = 1146 (53% male); mean age 42.78 ± 15.35 years;<br>99% native kidneys/ 1% transplanted kidneys	1D (diameter)	• < Minor bleeding <5 cm<br>• Middle bleeding >5 cm	Overall hemorrhage 37.7%: minor 29.4%, middle 7.3%, major 1.0% (defined as hemorrhage requiring transfusion / nephrectomy / death; not size-based); other discomfort symptoms 8.4%; no deaths	NO<br>All reported as subcapsular perinephric hematoma
**Demirelli et al. (2024) [58]**	US	Turkey<br>n = 259 (56% male); mean age 46 ± 14 years; 78% native kidneys / 22% transplanted kidneys	1D (exact measurement method not specified)	None defined	Major 5.8% (all transfusion; no embolization/surgery). Minor 8.5%. Additional 12.7% non-complicated hematoma; sizes up to 6 cm; no deaths	NO<br>Location NR at all
**Jung et al. (2025) [57]**	US	South Korea<br>n = 627 (58% male); mean age 51.3 ± 18.2 years;<br>native kidneys only	1D (maximal diameter)	Three size classes:<br>• ≤3 cm;<br>• >3–5 cm;<br>• >5 cm	Hematoma 69.7% (≤3 cm 56.1%, >3–5 cm 28.6%, >5 cm 15.3%); gross hematuria 3.8%; active bleeding 0.8%; transfusion 1.6%;	YES<br>• Perirenal<br>• Subcapsular

					embolization 1.0%; no surgery, no deaths.	
**Pinto-Silva et al. (2025) [54]**	US	Brazil n = 234 (51% male); mean age 48.0 ± 16.5 years; 98% native kidneys / 2% transplanted kidneys	3D (volumetric; exact measurement method not specified)	Hematomas >50 mL referred to emergency department	Hematomas up to 80 mL; 1 transfusion (0.43%); no embolization, no surgery, no deaths	NO<br><br>All reported as perirenal
**Murray et al. (2025) [55]**	US	Canada n = 580 (56% male); median age 49 (IQR 34–62) and 55 (IQR 38–66); 75% native kidneys / 25% transplanted kidneys	1D (diameter)	Large ≥5 cm	Bleed on US 14%; large hematoma >5 cm 3%; Hb drop >10 g/L 3%; AVF 0.1%; no surgery, no deaths	NO<br><br>Location NR at all
**Tsai et al. (2016) [64]**	US	Taiwan n = 269 (49% male); mean age 50.3 ± 12.8 years; transplanted kidneys only	2D (dimension x dimension; exact measurement method not specified)	None defined	Hematoma 1.1% (largest dimension up to 3 cm; all resolved); gross hematuria 2.2%; hydronephrosis 0.4%; Hb drop 0.7%; transfusion 0.7%; no AVF, no surgery, no death	NO<br><br>Location NR at all
**Chikamatsu et al. (2017) [32]**	CT	Japan n = 252 (61% male); mean age 62 ± 17 years; native kidneys only	3D (volumetric CT image segmentation)	Massive bleeding ≥85 mL (upper tertile)	Median bleeding volume 38 mL; macrohematuria 14.3%; hypotension 8.7%; transfusion 4.7%; bladder obstruction 1.6%; intervention 0.8%; no deaths	NO<br><br>Location not reported at all

Age is reported exactly as provided in each source publication. In most studies authors presented mean ± standard deviation (SD), whereas some reported mean with a range, and others reported non-normally distributed data, in which case the median with interquartile range (IQR, 25th–75th percentile) is cited. In rare cases mean age was not reported (NR) at all.

NR – nor reported; US – ultrasound; CT – computed tomography; 1D – one-dimensional measurement; 2D – two-dimensional measurement; 3D – three-dimensional volumetric measurement; Hb – hemoglobin; TXA – tranexamic acid; DDAVP – desmopressin; AVF – arteriovenous fistula; RBC – red blood cell; Hb – hemoglobin; NPV – negative predictive value.
